# Investigating the Role of T-bet^+^ B Cells (ABCs/DN) in the Immunopathogenesis of Systemic Lupus Erythematosus

**DOI:** 10.31138/mjr.34.1.117

**Published:** 2023-03-31

**Authors:** Athanasios Sachinidis, Maria Trachana, Anna Taparkou, George Gavriilidis, Panayotis Verginis, Fotis Psomopoulos, Christina Adamichou, Dimitrios Boumpas, Alexandros Garyfallos

**Affiliations:** 1 4^th^ Department of Internal Medicine, Hippokration General Hospital, School of Medicine, Aristotle University of Thessaloniki, Thessaloniki, Greece; 2 Paediatric Immunology and Rheumatology Referral Centre, 1^st^ Paediatric Department, Hippokration General Hospital, School of Medicine, Aristotle University of Thessaloniki, Thessaloniki, Greece,; 3 Institute of Applied Biosciences, Centre for Research and Technology Hellas, Thermi, Thessaloniki, Greece,; 4 Laboratory of Immune Regulation and Tolerance, Division of Basic Sciences, Medical School, University of Crete, Heraklion, Greece,; 5 4^th^ Department of Internal Medicine, “Attikon” University Hospital, National and Kapodistrian University of Athens, Athens, Greece

**Keywords:** ABCs, age-associated B cells, DN, SLE, T-bet, autoimmunity

## Abstract

**Background:**

Age-associated B cells (ABCs) constitute a B cell subset, defined as CD19^+^CD21^−^CD11c^+^, that expands continuously with age and accumulates strongly in individuals with autoimmune and/or infectious diseases. In humans, ABCs are principally IgD^−^CD27^−^ double-negative (DN) B cells. Data from murine models of autoimmunity, implicate ABCs/DN in the development of autoimmune disorders. T-bet, a transcription factor which is highly expressed in these cells, is considered to play a major role in various aspects of autoimmunity, such as the production of autoantibodies and the formation of spontaneous germinal centres.

**Aims of the study:**

Despite the available data, the functional features of ABCs/DN and their exact role in the pathogenesis of autoimmunity remain elusive. This project focuses on the investigation of the role of ABCs/DN in the pathogenesis of systemic lupus erythematosus (SLE) in humans, as well as the effects that various pharmacological agents may have on these cells.

**Methods:**

Samples from patients with active SLE will be used to enumerate and immunophenotype - via flow cytometry - the ABCs/DN found in the peripheral blood of the patients. Transcriptomic analysis and functional assays for the cells, both before and after in vitro pharmacological treatments, will also be performed.

**Anticipated benefits:**

The results of the study are expected to allow characterization of the pathogenetic role of ABCs/DN in SLE and could probably contribute, following careful association with the clinical state of the patients, towards the discovery and validation of novel prognostic and diagnostic markers of disease.

## BACKGROUND/RATIONALE

Transcription factor T-bet was first identified as a Th1 lineage commitment regulator.^1^ However, it soon became clear that it is also expressed in other cell types, such as NK cells, dendritic cells and B cells, and orchestrates their immune functions.^2,3,4^ In B cells, T-bet seems to be responsible for isotype switching to IgG2a (in mice) and the appearance of IgG2a-expressing memory B cells.^5,6^

Age-associated B cells (ABCs) is a CD19^+^CD21^−^CD11c^+^ B cell population that accumulates with age, and in autoimmune and infectious diseases, which has recently been described by several research groups.^7,8,9,10^ ABCs display an elevated expression of the transcription factor T-bet, as a result of a synergistic triggering of their BCR receptor, the TLR7 receptor and a third receptor, either IFNγR or IL21R.^8,11^ The exact role and the functions of these cells in aging, pathogenesis of autoimmunity and immune responses against pathogens are still elusive, and most intriguingly, some contrasting findings emerged.

Data from murine models of autoimmunity, implicate ABCs in the production of autoreactive IgG, the enhanced antigen presentation to T cells and the formation of spontaneous germinal centers.^8,12,13,14^ T-bet is considered to be the master regulator of all these processes,^12^ although recently published data, questions the importance of the transcription factor in the generation of functional ABCs.^15^

In humans, ABCs are mainly IgD^−^CD27^−^ double-negative (DN) B cells.^16,17,18^ DN have further been divided in two subgroups, based on the expression of CXCR5 chemokine, which serves as a follicular homing marker.^18^ The CXCR5+ subgroup (DN1) is expanded in the elderly healthy individuals and lacks T-bet expression, whereas the CXCR5^−^ subgroup (DN2) expresses T-bet and is more marked in systemic lupus erythematosus (SLE).^16,18,19^ The DN2 cells are hyper-responsive to TLR7 signalling and thus are poised to generate autoreactive antibody-secreting plasmablasts.^18^ In general though, their role in the development of autoimmunity remains elusive.

The expansion of ABCs/DN2 is pronounced in African-American SLE patients,^18^ a fact which indicates that genetic burden is a crucial factor for the generation of these cells. It is well known that ethnicity - among other factors - is linked to the severity of SLE manifestations,^20^ and a better understanding of the differences among the various ethnic groups could probably enable better management of the patients. To highlight this, it is important to mention that in a clinical trial, in which IFNγ was targeted via an anti-IFNγ monoclonal antibody, no therapeutic impacts were produced in a cohort of non-African-American SLE patients with lupus nephritis,^21^ probably due to the fact that ABCs/DN2 – which require IFNγ for their activation – are more marked in African-American patients. Taking into account these data, we find it important to assess whether SLE patient populations with different genetic backgrounds, such as a population from Greece, differs from an African-American population, in terms of ABC/DN2 immunophenotype.

## AIMS OF THE STUDY (OBJECTIVES)

There are three specific aims:

**1^st^ aim:** Enumerate and immunophenotype ABCs/DN in the peripheral blood of healthy donors and patients with active systemic lupus erythematosus (SLE), in order to identify correlations between the cell populations and the clinical profiles of the subjects. These correlations could evolve into useful prognostic and/or diagnostic markers of the disease.
**2^nd^ aim:** Evaluate the effects of “traditional” and “modern” pharmacological agents on ABC/DN’s percentage and functionality. The process shall be based on primary cell cultures combined with in vitro pharmacological treatments.
**3^rd^ aim:** Perform functional assays and transcriptomic analysis for ABCs/DN.

## RESEARCH STRATEGY (PATIENTS AND METHODS)

The project will be implemented in three work packages: Work package 1 deals with the 1^st^ aim of the project. We will be collecting blood samples from healthy individuals (n=15) and SLE patients (n=25). To be eligible for inclusion as a patient, the subject should not have been diagnosed with an active infectious disease and/or an autoimmune disorder, other than SLE. Moreover, as far as exclusion criteria are concerned, the treatments of the patients should not include biologics, as these pharmaceutical agents dramatically affect the B cell percentages. Please observe that the relatively small number of patients in our research protocol is dictated by the rarity of the disease. However, these rather small patient groups are common in the field and are in line with other published studies.

To enumerate and immunophenotype the T-bet+ B cells (ABCs/DN), we shall perform flow cytometric staining of whole blood samples. For this multi-parameter flow cytometric analysis, we will be using markers carefully selected from the literature, allowing characterization of both ABCs and DN. Despite the fact that, concerning DN cells, an emphasis will be placed on DN2 subset, as it is a discrete T-bet+ B cell population which is implicated in the pathogenesis of SLE,^18^ DN will be further examined for the T-bet, CD11c, CD21 and CXCR5 statuses, in order to investigate whether populations other than DN1 and DN2 exist in the individuals and probably play a role in SLE pathogenesis. The frequency detection of all these populations may contribute, following careful correlation with the clinical state of the subjects, towards the discovery and validation of novel prognostic and diagnostic markers of SLE.

Work package 2 will focus on the 2^nd^ aim, which refers to the evaluation of the effects that various pharmacological agents (may) have on ABCs/DN. The process shall be based on cell cultures combined with in vitro pharmacological treatments. Different concentrations of the agents will be used. We will be performing assays with both “traditional” molecules, such as hydroxychloroquine, and more “intelligent”, such as belimumab. Hydroxychloroquine is an antimalarial drug, which accumulates in the lysosomes, altering their pH and inhibiting several cellular processes. It is widely used in SLE, however its mechanism of action has not been fully elucidated and is believed to be associated with inhibition of TLR7 and TLR9 receptors and resulting signalling events, as well as the lysosomal degradation autophagic pathway.^22^ Belimumab, on the other hand, is a human monoclonal antibody, which binds with high affinity the B-cell activating factor BAFF, thus preventing BAFF interaction with its receptors (BR3, TAC1, BCMA) on the surface of B cells. Interaction of BAFF with its receptors is of vital importance for the survival and differentiation for B cells and belimumab was until recently the only biological agent approved for the treatment of SLE.^23,24^

To evaluate the effects of hydroxychloroquine, belimumab and others on T-bet^+^ B cells, we will be exposing primary cultures of whole blood (whole blood assay/WBA) or peripheral blood mononuclear cells (PBMCs) from SLE and healthy individuals to clinically relevant concentrations of the agents for varying periods and evaluate the effects on the T-bet^+^ B cell populations, using flow cytometry with ABC/DN markers. We believe that such an approach will provide direct evidence on the effects these agents have on T-bet^+^ B cells and could evolve into a valuable precision medicine tool, allowing prognosis of the patients’ response, even before the actual treatment begins. Moreover, these data could provide useful insight in the pathogenetic mechanisms of SLE and the involvement of T-bet^+^ B cells, thus contributing to the discovery and validation of novel druggable targets.

Work package 3 focuses on transcriptomic analysis and functional assays for ABCs/DN. The transcriptomic analysis shall be performed, at the single-cell level (scRNA seq), via a 10X Genomics platform,^25^ according to which a number of 10.000 cells per individual can be read. An SLE patient and a healthy donor will be examined. The results of the analysis are expected to allow characterisation of the genes and pathways associated with ABC/DN biology, paving the way for identification of novel genes of interest in SLE. With the aim of gaining as much sequencing information as possible for the ABCs and DN, cell sorting or magnetic beads isolation of CD19+ cells will be carried out prior to RNA isolation and sequencing. Of note, in order to examine more subjects than one patient and one healthy donor, we may alternatively (or additionally) perform a T-bet+ B cell transcriptomic meta-analysis. In such case, data shall be derived from already published scRNA seq studies.

As far as functional assays are concerned, we will use ELISA to estimate serum levels of cytokines (such as TNFα and IFNγ) and also autoantibodies. We will be collecting serum samples; along with the blood samples (see Work package 1). Moreover, we will use nephelometry to determine the levels of IgG isotypes. All these data shall then be used to identify possible correlations with the ABC/DN percentages.

All three work packages are expected to be completed until 2023.

## ANTICIPATED BENEFITS

In general, the results of the study are expected to allow characterization of the pathogenetic role of ABCs/DN in SLE and could probably contribute, following careful association with the clinical state of the patients, towards the discovery and validation of novel prognostic and diagnostic markers of the disease. Moreover, the elucidation of the role of ABCs/DN in the pathogenesis of SLE could lead to the discovery of novel druggable targets.

## ABBREVIATIONS

ABCs: age-associated B cells
DN: double-negative B cells
SLE: Systemic Lupus Erythematosus

## STUDY APPROVAL

The study has been approved by the Ethics Committee of Hippokration General Hospital, School of Medicine A.U.Th., Thessaloniki, Greece.
